# Does an Epidural Blood Patch (EBP) for Postdural Puncture Headache (PDPH) From a Lumbar Spinal Drain Necessitate a Distinct Approach?

**DOI:** 10.7759/cureus.35098

**Published:** 2023-02-17

**Authors:** Sindhuja Nimma, Adrian Maurer, Dimitrios Kampouri, Richa Wardhan

**Affiliations:** 1 Anesthesiology, Mayo Clinic, Jacksonville, USA; 2 Anesthesiology, University of Florida College of Medicine, Gainesville, USA

**Keywords:** postdural puncture headache, intracranial hypotension, epidural hematoma, subdural hematoma, cerebrospinal fluid leak, cerebrospinal fluid (csf), spinal drain, epidural blood patch

## Abstract

An intentional or unintentional dural puncture puts patients at significant risk for a postdural puncture headache (PDPH). When conservative treatments fail, an epidural blood patch (EBP) is offered cautiously due to rare but devastating complications. The literature is abundant with reviews on the management of PDPH in obstetric patients, but there is a paucity of data on the management of PDPH and complications of EBP in patients post spinal drain placement. In this case report, we address the specific concerns that vascular patients may have about the outcomes of large needle sizes and suggest alternative approaches for non-obstetric patients.

## Introduction

An intentional or unintentional dural puncture puts patients at risk for a postdural puncture headache (PDPH). The incidence of a PDPH depends on several factors including age, sex, body mass index (BMI), and needle type [1]. Specifically, in patients that undergo a spinal drain placement with a 14-gauge needle, the incidence of cerebrospinal fluid (CSF) leak and subsequent PDPH is much higher. The severity of symptoms from a CSF leak can range from self-resolving to agonizing positional headache and neurological symptoms. When conservative treatments fail or if the headache is significantly affecting activities of daily life, an epidural blood patch (EBP) is often offered cautiously as the first line of invasive treatment. In obstetric patients, symptomatic relief from the first EBP is approximately 50%-80% and approximately 90% with the second attempt, and complications, although rare, can be devastating [1,2]. Although the procedure itself is typically well-tolerated, the reported complications have ranged from back pain and exacerbation of headache to epidural hematoma, nerve palsy, arachnoiditis, and infection [1,2]. A rarer complication is the accidental injection of blood into the subdural or subarachnoid space [1,2] After obtaining formal written consent and Health Insurance Portability and Accountability Act (HIPAA) authorization, we discuss a case report of a patient who experienced a rare complication from an EBP post-spinal drain placement. Since there is a paucity of literature about offering an EBP to a patient with PDPH post spinal drain placement, we will delve into why careful evaluation is necessary prior to offering an EBP in non-obstetric patients.

## Case presentation

A 57-year-old male patient with a normal BMI presented to the emergency room (ER) with back pain and bilateral sciatica seven days after receiving an EBP. The patient with a history of abdominal aortic aneurysm had thoracic endovascular aortic repair (TEVAR) with a lumbar spinal drain placed at L4-5 prior to surgery. On postoperative day (POD) three, the lumbar drain was removed, and on POD-4, the patient started complaining of positional headaches fitting the description of a PDPH. When conservative therapy failed, coagulation status was confirmed with INR 1.2, and the patient was offered an EBP on POD-5. Under sterile conditions, the left antecubital vein was accessed, and an 18-gauge epidural needle was used to enter the epidural space at L4-5. Blood was injected in 10 mL increments, and ultimately, 40 mL of blood was required for symptomatic relief of headache. Placement of the spinal drain and the EBP were technically straightforward with no anatomical variations, and no back pain or paresthesias were noted immediately post-procedure. Two days after the EBP, the patient was discharged home.

**Figure 1:**
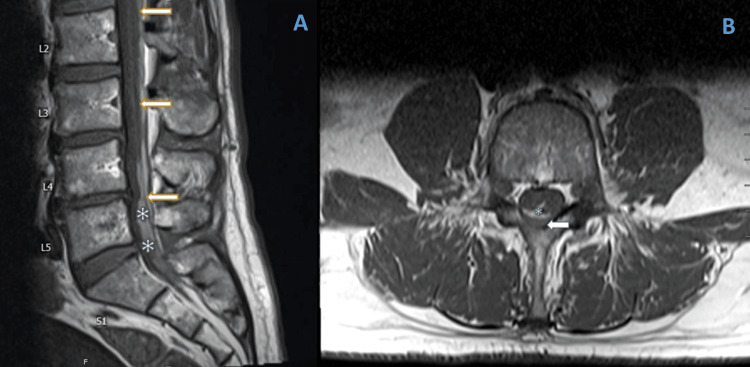
The T1-weighted sagittal (Panel A) and axial (Panel B) MR image of the spine demonstrates a subdural collection of blood (white arrows) extending from T12-L1 to sacral level and subarachnoid collection of blood (asterisks) extending from L3-4 to the termination of the thecal sac at S2.

One week after the EBP, the patient reported back to the ER with a fever (maximum temperature of 39.4°C), back pain, and excruciating bilateral sciatic pain that was progressively worsening. No urinary incontinence or saddle anesthesia was noted on the initial exam. The neurosurgery team was immediately consulted, and they recommended a spine magnetic resonance imaging (MRI) that showed a subdural collection of blood extending from T12-L1 to the sacral level and subarachnoid collection of blood extending from L3-4 to the termination of the thecal sac at S2 (Figure 1). Given the patient was also febrile on presentation, there was a suspicion of an epidural abscess, so blood cultures were drawn in the ER, which were negative. The neurosurgery team recommended nonoperative management with observation, pain control, and serial spine imaging. The patient was discharged the next day and followed up with the neurosurgery outpatient clinic. Over the next several weeks, the patient’s symptoms slowly improved, but he continued to have chronic back pain with no radiculopathy or saddle anesthesia. He was referred to a chronic pain clinic for his back and had significant relief after a medial branch and dorsal primary ramus nerve block. His most recent MRI, four months after his EBP, showed interval resolution of small-volume intradural hematoma within the dorsal spinal canal, which previously extended from T12-L1 to the sacrum. There was, however, clumping of cauda equina nerve roots at L4, L5, and S1, which suggests residual arachnoiditis. The patient, otherwise, is recovering slowly with no residual neurological deficits.

## Discussion

Patients undergoing major vascular surgery that could compromise perfusion of the spine may necessitate the placement of a spinal drain. As expected, any dural puncture, specifically one made with a large bore needle can put the patient at risk for persistent CSF leak leading to adverse effects such as PDPH, pseudomeningocele, spinal hematoma, CSF-cutaneous fistula, and meningitis [1,2]. Riley et al. demonstrated that a spinal drain placement carried an 18.3% incidence of PDPH, and conservative treatment was usually unsuccessful in this patient group [3]. When using a 25-gauge spinal needle, the incidence of PDPH was less than 1% but quickly increased to 36% with a 20/22-gauge needle, and the incidence doubled to more than 75% with a 17-gauge epidural needle [1]. As expected, when a 14-gauge needle is used to place a spinal drain, a large dural tear is likely created for CSF to escape and injected autologous blood to potentially enter the subdural and subarachnoid space.

An EBP is typically well-tolerated; however, reported complications have ranged from back pain and exacerbation of headache to epidural hematoma, nerve palsy, arachnoiditis, and infection. A rarer complication is the accidental injection of blood into the subdural or subarachnoid space, which could cause chemical or infectious meningitis, arachnoiditis, lower extremity weakness, paresthesias, or pneumocephalus [4]. Although transient back pain after EBP can occur in up to one-third of patients, if symptoms continue to worsen or if there is a newer onset of radiculopathy, there is a high suspicion for the presence of blood in the subdural or intrathecal space [1]. A recent case report of an obstetric patient who suffered from back pain and sacral radiculitis showed evidence of blood in the intrathecal space seven days after an EBP [5].

In the literature, there are numerous case reports of neuraxial complications after PDPH, but almost all are reported in obstetric patients where the inciting event occurred from a 17-18-gauge needle in mostly healthy patients [4,6-8]. Of the same case reports describing neuraxial complications, half reported autologous blood in the intrathecal space and the rest in the subdural space. No case report describes the presence of blood in both the intrathecal and subdural spaces combined. In one case, a hematoma was seen both in the subdural and subarachnoid space, which led to severe back pain and radiculopathy six days after the EBP [5].

Although there is no optimal safe volume of blood that can be injected into the epidural space during an EBP, the American Society of Anesthesiologists recommends using 20 mL of blood and stopping the injection before 20 mL if not tolerated by the patient [1]. After reviewing literature that studied low versus high volumes of autologous blood injectate in obstetric patients, the incidence of permanent headache relief was highest in the 20 mL group [9]. Although theoretically, there is a higher chance of a large volume of autologous blood entering the subdural/intrathecal space with a larger dural leak, there are no substantiated recommendations about the safe volume of autologous blood that can be injected for a PDPH post-spinal drain removal. In the current case presented, autologous blood was injected in 10 mL increments until the patient experienced fullness in his back as well as symptomatic relief of headache at 40 mL. There is certainly a potential risk associated with continued injection of blood beyond 20 mL; however, given no predetermined maximum recommendation or symptomatic relief, the patient was assessed after every 10 mL injection until 40 mL of blood was ultimately injected.

An EBP in obstetric patients is typically performed either at the same level as the dural puncture or one to two levels below because blood injected during an EBP spreads predominantly cranially. Performing an EBP at the same spinal level as the spinal drain can reintroduce skin bacteria with direct access to the subdural structures, given large dural tears. Central line studies looking at the reinsertion of a new catheter over a guide wire indicated that the puncture channel was often colonized by the microorganisms on the outside of the previous catheter, thereby increasing the risk of transfer of bacterial colonization. Extraluminal colonization occurs frequently from the catheter puncture site [10]. Another study by Tsai et al. looked at 30 cases with reported CSF-cutaneous fistulas, and nine of the cases involved the use of 14-gauge or larger needles [11]. Several risk factors were hypothesized to contribute to the development of a CSF-cutaneous fistula, which included the use of steroids, multiple passes at the same spinal level, duration of the subarachnoid drainage, and catheter size. Multiple passes at the same spinal level may also lead to fibrin deposition along the tract, which would interfere with tract closure [11,12]. Although re-introduction of the blood patch needle through the same spinal level is acceptable for EBP in obstetric patients, perhaps in patients with the possibility of a CSF-cutaneous fistula and delayed healing, it would be more prudent to perform the EBP at a spinal level away from the original insertion site. This could also help reduce the infection rate, the risk of blood entering the subdural/intrathecal space, and the subsequent neurological sequelae.

This case report raises several critical points. First and foremost, it is important to note that complications associated with a lumbar spinal drain placement can be severe even when performed by a dedicated team of experts. If any difficulty is anticipated in performing the EBP, imaging and fluoroscopy may help identify the epidural spread of blood. If patients complain of radiculopathy, immediately consider subarachnoid hematoma as a complication until it can be ruled out with MRI studies. Though not commonly employed at many institutions, there are alternatives to failed EBPs such as non-blood containing epidural injections, fibrin glue seal, sphenopalatine nerve block, and various oral and intravenous medications that could perhaps be considered as a secondary option [13].

Informed consent as well as detailed discharge instructions are vital to every conversation because complications from an EBP, although rare, can be devastating. There is a paucity of literature review about the guidelines for vascular patients undergoing an EBP from a large-needle PDPH, and perhaps further studies can elucidate some of these concerns. For our patient, the rare complication of a subdural/intrathecal hematoma was identified quickly; imaging studies were performed in a timely manner, and the neurosurgery team was immediately involved for further evaluation. Although symptoms gradually improved without requiring surgical intervention, escalation of care was critical.

## Conclusions

An intentional or unintentional dural puncture puts patients at significant risk for a PDPH. For a procedure like a spinal drain requiring a 14-gauge introducer needle, perhaps, the risk of PDPH is higher, and the complications of EBP as a treatment may come with rare and devastating complications. We aimed to address these specific concerns and hope that other physicians reading this case report will be more cautious in not only offering patients EBPs but also appropriately following up on such rare complications.
